# Inflammatory potential of the diet and risk of sarcopenia and its components

**DOI:** 10.1186/s12937-020-00649-2

**Published:** 2020-11-28

**Authors:** Amir Bagheri, Sanaz Soltani, Rezvan Hashemi, Ramin Heshmat, Ahmadreza Dorosty Motlagh, Ahmad Esmaillzadeh

**Affiliations:** 1 Students’ Scientific Research Center, Tehran University of Medical Sciences, Tehran, Iran; 2 Department of Community Nutrition, School of Nutritional Sciences and Dietetics, Tehran University of Medical Sciences, P.O. Box 14155-6117, Tehran, Iran; 3 Department of Geriatric Medicine, Ziaeian Hospital, Tehran University of Medical Sciences, Tehran, Iran; 4 Chronic Diseases Research Center (CDRC), Endocrinology and Metabolism Population Sciences Institute, Tehran University of Medical Sciences, Tehran, Iran; 5 Obesity and Eating Habits Research Center, Endocrinology and Metabolism Molecular -Cellular Sciences Institute, Tehran University of Medical Sciences, Tehran, Iran; 6 Department of Community Nutrition, Isfahan University of Medical Sciences, Isfahan, Iran

**Keywords:** Dietary inflammatory index, Sarcopenia, Muscle mass, Muscle strength

## Abstract

**Background:**

Despite a large body of evidence on the link between dietary inflammatory index (DII) and several chronic conditions, limited data are available about the association of DII and sarcopenia. This study aimed to examine the relationship between inflammatory potential of the diet (as measured by DII) and sarcopenia and its components among community-dwelling elderly population.

**Methods:**

This population-based cross-sectional study was performed in 2011 among 300 elderly people (150 men and 150 women) aged ≥55 years, who were selected using cluster random sampling method. Dietary assessment was done using a pre-tested food frequency questionnaire. Energy-adjusted DII was calculated based on earlier studies. Sarcopenia and its components were determined based on the European Working Group on Sarcopenia (EWGSOP) definition.

**Results:**

Mean age of study participants was 66.7 ± 7.7 y. Subjects in the highest tertile of DII score (i.e. those with a more pro-inflammatory diet) were more likely to be older (*P* = 0.02). The prevalence of sarcopenia (*P* = 0.016) and low muscle mass (*P* = 0.041) was significantly higher among subjects in the top tertile compared with those in the bottom tertile of DII. After adjustment for potential confounders, those with the highest DII were 2.18 times (95% CI: 1.01–4.74) more likely to have sarcopenia than those with the lowest DII. With regard to components of sarcopenia, subjects in the top tertile of DII had not significantly greater odds of low muscle mass (OR: 1.38; 95% CI: 0.72–2.63), abnormal handgrip strength (OR: 0.97; 95% CI: 0.49–1.89), and abnormal gait speed (OR: 1.61; 95% CI: 0.84–3.08) than those in the bottom tertile.

**Conclusions:**

In conclusion, a diet with more pro-inflammatory potential was associated with a greater odds of sarcopenia. Further studies are required to confirm these findings.

## Introduction

Sarcopenia is described as a geriatric syndrome determined by wasting muscle mass in addition to reduced muscle strength and/or physical performance [1]. Although sarcopenia is common in older adults, it can also occur early in life [2]. The reported prevalence of sarcopenia varies greatly depending on the population studied and the definition criteria, from 1 to 29% in elderly community-dwelling populations in western countries [3] and 2 to 46% in Asia [4]. This disorder is associated with higher rates of falls, decreased function, high mortality rates, and increased probability of hospitalization [5, 6].

Inflammation is a major risk factor for several conditions in the elderly. Inflammatory cytokines lead to rapid muscle wasting, eventually stimulate protein catabolism and suppress muscle synthesis [7]. Dietary factors have been shown to contribute to inflammation [8]. High intakes of fruits and vegetables [9], and also specific nutrients such as fiber [10], omega-3 fatty acids [11], vitamin E [12], and vitamin C [13] were associated with lower concentrations of circulating inflammatory markers. To determine the inflammatory potential of the whole diet, Dietary Inflammation Index (DII) has been designed and validated against circulating levels of inflammation [14, 15]. This dietary index has linked with several cancers, metabolic diseases, and fractures [16–18]. Two longitudinal cohort studies have also shown that higher DII scores were associated with a higher incidence of frailty [19] and decreased appendicular lean mass [8]. Given the limited studies on the association of inflammatory potential of the diet with muscle health, along with the great differences in dietary intakes, lifestyle factors and body composition of people in the Middle East with those in Western countries [20–22], this cross-sectional study aimed to investigate the relationship between DII with sarcopenia in an Iranian population.

## Materials and methods

### Participants

This population-based cross-sectional study was performed from May to October 2011 in Tehran, Iran. The detailed report on the sampling method and data collection procedure has been published previously [23]. In total, 300 elderly people (150 men and 150 women) with aged ≥55 years were enrolled by the use of cluster random sampling method in district 6 of Tehran. The head of each cluster was selected based on a ten-digit postal code. To ensure the homogeneity of our sample, individuals whose potential cause of sarcopenia were factors other than aging were not invited. Indeed, people who were susceptible to sarcopenia due to secondary causes [24], including those who were unable to move, subjects with artificial limbs or limb prostheses, or individuals with debilitating diseases that predispose the person to sarcopenia (e.g malignancy, organ failure) were not included in the study.

### Dietary intake assessment

Usual dietary intakes of the study participants was assessed using a 117-item Food Frequency Questionnaire (FFQ); the validity and reliability of this questionnaire was reported in previous studies [23, 25]. The questionnaire consisted of a list of foods with a specific portion size. Participants were able to report their consumption frequency based on daily, weekly or monthly basis for each food item. The questionnaire was filled by a trained nutritionist through face-to-face interview. After completing the FFQ, the frequency of each food item was converted to grams per day considering the household measures of portion sizes. Daily energy and nutrients intake of each participant was calculated by using Nutritionist IV software with a modified food composition database based on the US Department of Agriculture.

### Construction of dietary inflammatory index

The method of Shivappa et al. was used to compute the Dietary Inflammatory Index (DII) [14]. DII score was calculated using 29 dietary parameters because some parameters suggested in the original scoring method were not available in our dataset in Iran. Dietary parameters included: energy, carbohydrate, fat, protein, fiber, cholesterol, mono-unsaturated fatty acids (MUFAs), polyunsaturated fatty acids (PUFAs), saturated fats (SFAs), vitamin B12, pyridoxine (B6), folic acid, niacin (B3), riboflavin (B2), thiamin (B1), vitamin A, C, D, E, b-carotene, zinc, selenium, magnesium, iron, caffeine, pepper, onion, garlic and green/black tea. Residual method was used to obtain the energy-adjusted amounts for all nutrients [26]. Then, to get z-score, the “standard global mean” was subtracted from the quantity of food and it was divided by the “global standard deviation”. Standard global means and SDs for each dietary item were obtained from Shivappa et al. [14]. To decrease skewness, this value was converted to a centered percentile score. Then, this score was multiplied by the effect score for each of the food items obtained from Shivappa et al. [14]. Finally, to compute a total DII score for each participant, we summed the DII score obtained from all individual dietary parameters. The most negative score implies the maximum anti-inflammatory diet, while the most positive score implies the maximum pro-inflammatory diet.

### Assessment of sarcopenia

Based on the European Working Group on Sarcopenia (EWGSOP) definition [24], sarcopenia was determined by considering the combination of both low muscle mass and low muscle function (either strength or performance). The muscle mass was measured as the ratio of an individual’s total lean mass of legs and arms (also named Appendicular Skeletal Muscle or ASM) [27] to their squared height (ASM/height^2^). ASM was calculated with a DXA scanner (Discovery W S/N 84430). Based on EWGSOP, low muscle mass was considered as the amount of muscle mass less than 5.45 kg/m^2^ for women and 7.26 kg/m^2^ for men [24].

A handgrip test was used to measure muscle strength. The handgrip test was assessed by a pneumatic instrument that is a squeeze bulb dynamometer (c7489–02 Rolyan) calibrated in pound per square inch (psi). The handgrip strength (maximum voluntary contractions) was calculated three times for each right and left hand with a 30-s rest between measurements. Then, the average of these three measurements for each hand was calculated. Finally, the average number was obtained based on the sum of mean of both hands and this was considered as muscle strength. Sex and age-specific cutoff points recommended by Merkies et.al was then used to identify low muscle strength [28]. To measure muscle performance, a 4-Meter walk gait speed test was applied [24]. Participants who had gait speeds less than 0.8 m/s were recognized as low muscle performance [24].

### Assessment of other variables

Information about general characteristics of participant including age, sex, socio-economic status, medical history, medication use, smoking habits, and alcohol consumption were collected by a pre-tested questionnaire. The physical activity level in this study was examined by a trained interviewer using the short form of the International Physical Activity Questionnaire (IPAQ), its validity has previously been examined [25]. Measures of physical activity for each participant was expressed as metabolic equivalent-hour per week (MET-h/week) based on IPAQ’s guideline [29]. Weight was measured using a digital scale while participants were minimally clothed. Height was measured by a wall tape meter in standing position without shoes. Waist circumference was measured in the middle of the lower rib margin and iliac crest while participants were stand up and normally breathe. Weight (kg) divided by height squared (m^2^) was used to calculate body mass index (BMI).

### Statistical analysis

Subjects were classified according to the tertiles of DII score. General characteristics of study participants across tertiles of DII score were compared using Chi-square for categorical variables and ANOVA for continuous variables. Age- sex-, and energy-adjusted dietary intakes of participants were computed using General Linear Model and compared using ANCOVA across tertile categories of DII score. Multivariable logistic regression was conducted to find the relationship between inflammatory potential of the diet and odds of sarcopenia. In these analyses, several confounders were controlled. First, the association was adjusted for age (continuous), sex (male/female) and energy intake (kcal/d). Then, further controlling was done for physical activity (MET-h/wk), smoking (yes/no), alcohol consumption (yes/no), medication use (statin, corticosteroid, estrogen, testosterone), and positive history of chronic disease means subjects that affected by one of the following diseases: asthma, arthritis, myocardial infarction, cerebrovascular accident, and diabetes. In all these analyses, the bottom tertile was considered as the reference category and the odds ratio for sarcopenia in other categories was calculated. To evaluate the linear trend across tertiles of the DII score, the tertile categories were considered as an ordinal variable in the models. All analyses were done using SPSS (version 26). *P*-values were considered significant at < 0.05.

## Results

General characteristics of study participants across tertile categories of DII score are provided in Table 1. Subjects in the highest tertile were more likely to be older (*P* = 0.02) and less likely to be female (*P* = 0.008) than those in the lowest tertile of the DII score. The DII range was - 3.98 to 4.29, and participants in top tertile of DII had higher DII score indicating more pro-inflammatory diet than those in the bottom (*P* <  0.001). There was no other significant difference between the lowest and highest tertiles of DII in terms of mean BMI and distribution of participants in terms of alcohol use, smoking, medication use and disease history.

**Table 1:** Characteristics of study participants in tertile categories of DII score ^a^

	Tertiles of DII score			*P*-value ^*b*^
	T_1_ (*n* = 100)	T_2_ (*n* = 100)	T_3_ (*n* = 100)	
Range of DII	<−0.88	−0.88, − 0.70	> − 0.71	
DII	−1.95 ± 0.74	−0.03 ± 0.46	1.98 ± 0.78	< 0.001
Age (y)	66.55 ± 7.47	65.47 ± 7.01	68.37 ± 8.31	0.02
BMI (kg/m^2^)	27.58 ± 4.32	26.91 ± 3.64	27.64 ± 4.60	0.39
Physical activity (MET-h/w)	1481.55 ± 1464.81	1170.93 ± 1098.09	1231.10 ± 1663.12	0.26
Female (%)	62	51	40	0.008
Alcohol use (%)	9	17	14	0.243
Smoking (%)	7	14	17	0.093
Medical history ^c^				0.739
Yes (%)	15	18	19	
No (%)	85	82	81	
Drug history				
Sexual hormone use (%)	6	1	2	0.090
Statin use (%)	43	32	35	0.248
Corticosteroid use (%)	2	3	3	0.879

^a^ All values are mean ± SD, unless indicated; ^b^ ANOVA for continuous variables and Chi-squared test for categorical variables

^c^ Medical history Yes means patients with one of the following diseases: asthma, arthritis, myocardial infarction, cerebrovascular accident, diabetes

*DII*: Dietary Inflammatory index

Table 2 presents age-, gender- and energy-adjusted dietary intakes of study participants across tertile categories of DII. Individuals in the highest tertile of DII had higher intakes of SFA, and lower intake of protein, iron, magnesium, zinc, thiamine, riboflavin, niacin, vitamin B6, b-carotene, vitamin A, vitamin C, dietary fiber, folate, pepper, onion, and garlic compared with those in the lowest tertile (all *P*-values were <  0.05).

**Table 2:** Dietary intakes of study participants by tertile categories of DII score ^a^

	Tertiles of DII score			
	T_1_ (*n* = 100)	T_2_ (*n* = 100)	T_3_ (*n* = 100)	***P***-value ^***b***^
Range of DII	<−0.88	−0.88, −0.70	> − 0.71	
Energy (kcal/d)	2262 ± 92.36	2153 ± 92.05	2373 ± 92.93	0.25
Nutrients				
Carbohydrates (g/d)	368.32 ± 5.51	366.63 ± 5.50	363.12 ± 5.56	0.799
Proteins (g/d)	89.55 ± 1.78	86.98 ± 1.78	81.66 ± 1.80	0.008
Total fats (g/d)	58.15 ± 1.89	58.37 ± 1.89	61.33 ± 1.91	0.43
Cholesterol (mg/d)	194.32 ± 7.95	195.94 ± 7.94	218.16 ± 8.02	0.068
SFA (g/d)	16.72 ± 0.53	17.51 ± 0.53	19.24 ± 0.53	0.004
MUFA (g/d)	19.24 ± 0.83	18.88 ± 0.83	19.62 ± 0.83	0.824
PUFA (g/d)	14.02 ± 0.77	14.15 ± 0.77	14.65 ± 0.78	0.838
Fe (mg/d)	21.53 ± 0.32	20.08 ± 0.32	18.48 ± 0.32	< 0.001
Mg (mg/d)	483.76 ± 8.62	450.09 ± 8.61	385.48 ± 8.69	< 0.001
Zn (mg/d)	12.65 ± 0.28	12.62 ± 0.28	11.64 ± 0.28	0.022
Se (mg/d)	0.097 ± 0.003	0.097 ± 0.003	0.098 ± 0.004	0.965
Thiamine (mg/d)	2.31 ± 0.04	2.28 ± 0.04	2.09 ± 0.04	0.001
Riboflavin (mg/d)	2.56 ± 0.05	2.39 ± 0.05	2.20 ± 0.05	< 0.001
Niacin (mg/d)	21.91 ± 0.37	21.21 ± 0.37	20.16 ± 0.37	0.005
Vitamin B6 (mg/d)	2.97 ± 0.12	2.71 ± 0.12	2.14 ± 0.12	< 0.001
b-carotene (mcg/d)	3411.3 ± 97.60	2265.7 ± 97.51	1365.8 ± 98.44	< 0.001
Vitamin A (RE/d)	2468.6 ± 60.76	1709.5 ± 60.70	1244.9 ± 61.28	< 0.001
Vitamin C (mg/d)	355.26 ± 8.51	268.67 ± 8.50	201.12 ± 8.58	< 0.001
Vitamin E (mg/d)	9.69 ± 0.5	9.17 ± 0.5	9.40 ± 0.5	0.760
Vitamin D (mcg/d)	2.38 ± 0.19	1.85 ± 0.19	1.81 ± 0.19	0.069
Vitamin B12 (mcg/d)	4.78 ± 0.23	4.65 ± 0.23	4.23 ± 0.24	0.240
Folate (mcg/d)	617.99 ± 9.65	553.81 ± 9.64	458.84 ± 9.73	< 0.001
Dietary fiber (g/d)	36.18 ± 0.70	29.53 ± 0.70	24.24 ± 0.71	< 0.001
Pepper	25.59 ± 2.69	12.36 ± 2.69	7.17 ± 2.71	< 0.001
Onion	42.22 ± 3.17	32.53 ± 3.17	24.81 ± 3.20	0.001
Garlic	4.04 ± 0.51	2.09 ± 0.51	1.89 ± 0.51	0.006
Green/Black tea	757.89 ± 56.85	824.39 ± 56.80	754.70 ± 57.34	0.578
Caffeine	177.54 ± 11.93	182.46 ± 11.89	162.47 ± 11.98	0.477

^a^ All values are mean ± SE; energy intake is adjusted for age and sex, all other values are adjusted for age, sex and energy intake

^b^ ANCOVA for all variables

The prevalence of sarcopenia and its components across tertile categories of DII are shown in Table 3. Prevalence of low muscle mass and sarcopenia were significantly higher among subjects in the top tertile of DII than those in the bottom tertile (*P* = 0.041). There were no significant differences between means of muscle mass, handgrip strength and gait speed across tertile categories of DII after adjusted for age, sex, and energy.

**Table 3:** Prevalence of sarcopenia and its components across tertile categories of DII score

	Tertiles of DII diet score			*P*-value ^a^
	T_1_ (*n* = 100)	T_2_ (*n* = 100)	T_3_ (*n* = 100)	
Range of DII	<−0.88	− 0.88, − 0.70	> − 0.71	
Prevalence of components of sarcopenia				
Low muscle mass (%) ^b^	33	35	49	0.041
Abnormal hand grip strength (%) ^c^	38	28	30	0.276
Abnormal gait speed (m/s) (%) ^d^	34	44	44	0.251
Sarcopenia (%)	14	13	27	0.016
Means of components				
Muscle mass [ASM/h2] (kg)				
Crude	6.52 ± 1.02	6.61 ± 1.00	6.68 ± 0.94	0.526
Model ^1^	6.65 ± 0.08	6.59 ± 0.08	6.58 ± 0.08	0.814
Hand grip strength (psi)				
Crude	10.31 ± 3.04	11.60 ± 3.88	11.21 ± 3.65	0.031
Model ^1^	10.84 ± 0.23	11.42 ± 0.23	10.87 ± 0.23	0.145
Gait speed (m/s)				
Crude	0.86 ± 0.20	0.84 ± 0.23	0.82 ± 0.23	0.609
Model ^1^	0.87 ± 0.02	0.83 ± 0.02	0.82 ± 0.02	0.327

^a^ Obtained from ACNOVA for quantitative variables and chi-square for qualitative variables (*P* < 0.05 significant)

^b^ Muscle mass lower than 5.45 (kg/m2) for women and 7.26 (kg/m2) for men were considered low [24]

^c^ Abnormal muscle strength was defined according previous study [28]

^d^ Gait speeds lower than 0.8 m/s were considered abnormal [24]. Model^1^: Adjusted for energy, age and sex

Findings from multivariable logistic regression analysis indicating the association between inflammatory potential of diet and odds of sarcopenia are presented in Table 4. In the crude model, it was found that subjects in the highest tertile of DII had a higher likelihood of sarcopenia (OR: 2.27; 95% CI: 1.10–4.65) compared with those in the lowest tertile. When the analysis was controlled for age, sex, and energy intake, participants with the greatest DII score had higher odds of sarcopenia (OR: 2.01; 95% CI: 0.96–4.19) than those with the lowest DII, however, this was not statistically significant. Further adjustments for other covariates revealed that subjects in the top tertile of DII were 2.18 times (95% CI: 1.01–4.74) more likely to have sarcopenia than those in the bottom tertile.

**Table 4:** Multivariable-adjusted odds ratios (95% CIs) for sarcopenia and its components across tertile categories of DII score

	Tertiles of DII diet score			*P* for trend
	T_1_	T_2_	T_3_	
Range of DII	<−0.88	−0.88, − 0.70	> − 0.71	
Sarcopenia				
N	100	100	100	
Crude	1	0.91 (0.40–2.06)	2.27 (1.10–4.65)	0.018
Model 1	1	0.91 (0.40–2.07)	2.01 (0.96–4.19)	0.051
Model 2	1	1.01 (0.43–2.39)	2.18 (1.01–4.74)	0.035
Low muscle mass ^a^				
Crude	1	1.09 (0.60–1.96)	1.96 (1.10–3.46)	0.021
Model 1	1	0.99 (0.53–1.85)	1.46 (0.79–2.71)	0.219
Model 2	1	0.98 (0.51–1.88)	1.38 (0.72–2.63)	0.312
Abnormal hand grip strength ^b^				
Crude	1	0.63 (0.35–1.15)	0.69 (0.38–1.25)	0.226
Model 1	1	0.69 (0.36–1.29)	0.98 (0.51–1.86)	0.901
Model 2	1	0.66 (0.34–1.29)	0.97 (0.49–1.89)	0.902
Abnormal gait speed (m/s) ^c^				
Crude	1	1.52 (0.86–2.70)	1.52 (0.86–2.70)	0.151
Model 1	1	1.88 (1.02–3.46)	1.78 (0.96–3.29)	0.064
Model 2	1	1.81 (0.95–3.45)	1.61 (0.84–3.08)	0.157

Model 1: Adjusted for age, sex and energy intake. Model 2: Further adjusted for physical activity, smoking, alcohol consumption, medication use (statin, corticosteroid, estrogen, testosterone), and positive history of disease (asthma, arthritis, myocardial infarction, cerebrovascular accident, diabetes)

^a^ Muscle mass lower than 5.45 (kg/m2) for women and 7.26 (kg/m2) for men were considered low [24]

^b^ Abnormal muscle strength was defined according previous study [28]

^c^ Gait speeds lower than 0.8 m/s were considered abnormal [24]

Furthermore, when the analyses were conducted for components of sarcopenia, we found that subjects in the top tertile of DII had a higher odds of low muscle mass (OR: 1.96; 95% CI: 1.10–3.46) compared with those in the bottom tertile; however, this association disappeared when potential confounders were taken into account (OR: 1.38; 95% CI: 0.72–2.63). There was no significant association between DII and abnormal hand grip strength as well as abnormal gait speed after controlling for covariates.

## Discussion

Our findings indicated that increased DII score, indicating a more pro-inflammatory diet which is related to greater serum inflammatory markers, was associated with an increased odd of sarcopenia in older adults. This association remained significant even after adjustment for potential confounders. However, no significant association was seen between DII and components of sarcopenia including low muscle mass, abnormal handgrip strength, and abnormal gait speed. To our knowledge, this is the first study investigating the association between the inflammatory potential of the diet and sarcopenia.

In the current study population, total DII score ranged from − 3.98 to 4.29. We also found a positive association between higher DII scores and odds of sarcopenia. Although some studies had reported the relationship between DII and muscle mass and muscle strength, data on sarcopenia are limited. In a longitudinal cohort study in the US adults, DII was investigated in relation to frailty (defined as containing two of these criteria: weight loss, inability to rise from a chair 5 times, and poor energy). They reported that the range of DII in their participants was − 5.65 to + 3.70 and subjects in top quartile of DII score had a higher incidence of frailty in men [19]. Findings from a study on 1344 postmenopausal Korean women aged 50 years or older, revealed that the DII score range was − 6.37 to + 6.55, and no significant association was found between higher DII score (higher versus lower than - 0.07) and osteosarcopenic conditions after adjustment for potential confounders [30]. Despite a positive association with sarcopenia, we failed to find any significant association between DII and individual components of sarcopenia including low muscle mass, abnormal handgrip strength and abnormal gait speed. A prospective population-based study in Australia with 1099 men and women aged 50–79 years old revealed that the range of DII was − 3.80 to + 3.23 and each unit increase in DII score was not associated with a lower appendicular lean mass and handgrip strength after controlling for covariates [8]. It seems that additional information is required to shed light on the link between dietary inflammatory index and individual components of sarcopenia, given that there is a possible association between inflammation and sarcopenia, as indicated in a recent meta-analysis [7]. Low-grade chronic inflammation seems to predispose elderly people to muscle loss and dysfunction through affecting muscle proteolysis and myocyte apoptosis [31, 32]. Therefore, it is logical to expect finding an association between inflammatory potential of the diet and sarcopenia and its components.

Another finding of our study is that subjects in the third tertile of sarcopenia had significantly lower intakes of the anti-inflammatory components of DII including protein, iron, magnesium, zinc, thiamine, riboflavin, niacin, vitamin B6, b-carotene, vitamin A, vitamin C, folate, dietary fiber, pepper, onion, garlic than those in the first tertile. Moreover, participants in the top tertile had higher intakes of saturated fatty acids than those in the bottom tertile. This finding indicates that individuals with the greater adherence to a pro-inflammatory diet have lower intakes of fruit, vegetables, and protein and higher intakes of unhealthy fat. Based on the current dietary recommendations to live longer, high consumption of fruit and vegetables has been suggested [33–35]. A prospective cohort study among people aged ≥65 years in Hong Kong showed that greater adherence to “vegetables-fruits” pattern and “snacks-drinks-milk products” pattern was associated with a lower odds of sarcopenia in men [5]. In the Newcastle 85+ cohort study, the investigators reported a positive association between a “traditional British” dietary pattern, characterized by high intakes of butter, red meat, gravy, and potato, and sarcopenia [36]. Existing evidence suggests that increased inflammation and production of pro-inflammatory cytokines (e.g CRP, IL-6, TNF-a) are associated with lower muscle mass, muscle strength, and physical performance [8]. The plausible mechanism might be the role of inflammatory cytokines in declining anabolic factors such as insulin and insulin-like growth factor-1 (IGF-1) through which they can down-regulate muscle protein synthesis [37]. Therefore, high intake of dietary factors with anti-inflammatory properties may have beneficial effects on sarcopenia.

This study has several strengths. Several potential confounders were controlled for in the current analysis. Furthermore, a validated FFQ was used for the assessment of dietary intakes. The main limitation of our study is its cross-sectional design, which prohibits determining a causal relationship. Another limitation is the misclassification of study participants and measurement error due to the use of the FFQ. To reduce the effect of these errors, energy-adjusted DII was used in our study. Because of the limited data on some components of DII such as n-3 and n-6 fatty acids, eugenol, thyme/oregano, saffron, turmeric, trans fat, ginger, rosemary, and flavonoids, we did not include them in the DII calculation that may have caused an underestimation of the association; however, Shivapa et al. [14] showed that including at least 28 dietary parameters for calculating DII did not reduce the predictive ability of DII. Although the association between DII and inflammatory factors has been validated in previous studies [14], no information on serum inflammatory markers were available in the current study to examine its accuracy in the current population. In addition, it must be kept in mind that the current scoring of DII, as suggested by Shivappa et al. [14], was constructed based on available literature on diet and inflammation before 2010; however, a large body of evidence on the link between dietary factors and inflammation came out since that time. Therefore, it seems that a new updated scoring method, which take the current literature into account, must be developed. Lastly, the study was conducted on a small sample from the limited area where the DEXA device was located, because of financial limitations and inadequate access to the DEXA device in Tehran (maximum 300 cases). Accordingly, the generalization of these results to the whole Iranian population should be done with caution. Also, it is important to note that the current study was conducted on older adults. Therefore, extrapolation of the present findings to other age groups should be done cautiously.

## Conclusion

In conclusion, greater adherence to a diet with more pro-inflammatory properties was associated with increased odds of sarcopenia in older adults. These findings support the current dietary recommendations on reducing the consumption of unhealthy foods with pro-inflammatory properties to reduce the risk of several chronic conditions including sarcopenia. Further well-designed studies are required to investigate and clarify these associations.

## Data Availability

The data are not publicly available.

## Abbreviations

DII: Dietary Inflammatory Index
OR: Odds Ratio
CI: Confidence Interval
SD: Standard Deviation
FFQ: Food Frequency Questionnaire
IPAQ: International Physical Activity Questionnaire
MET-h/week: Metabolic Equivalents-hours per week
BMI: Body Mass Index
SFA: Saturated Fatty Acid
MUFAs: mono-unsaturated fatty acids
PUFAs: polyunsaturated fatty acids
EWGSOP: European Working Group on Sarcopenia
ASM: Appendicular Skeletal Muscle
PSI: Pound per Square Inch
